# Direct comparison of Hoxb8-driven reporter distribution in the brains of four transgenic mouse lines: towards a spinofugal projection atlas

**DOI:** 10.3389/fnana.2024.1400015

**Published:** 2024-05-16

**Authors:** Bridget N. Barraclough, W. Terrence Stubbs, Manon Bohic, Aman Upadhyay, Victoria E. Abraira, Matt S. Ramer

**Affiliations:** ^1^International Collaboration on Repair Discoveries, The University of British Columbia, Vancouver, BC, Canada; ^2^Department of Zoology, The University of British Columbia, Vancouver, BC, Canada; ^3^W.M. Keck Center for Collaborative Neuroscience, Rutgers, The State University of New Jersey, Piscataway, NJ, United States; ^4^Department of Cell Biology and Neuroscience, Rutgers, The State University of New Jersey, Piscataway, NJ, United States

**Keywords:** spinofugal tract, spinothalamic tract, spinotectal tract, spinocerebellar tract, lineage tracing, sensory systems, Hoxb8

## Abstract

**Introduction:**

Hox genes govern rostro-caudal identity along the developing spinal cord, which has a well-defined division of function between dorsal (sensory) and ventral (motor) halves. Here we exploit developmental Hoxb8 expression, normally restricted to the dorsal cord below the obex, to genetically label spinal cord-to-brain (“spinofugal”) axons.

**Methods:**

We crossed two targeted (knock-in) and two non-targeted recombinase-expressing lines (Hoxb8-IRES-Cre and Hoxb8-T2AFlpO; Hoxb8-Cre and Hoxb8-FlpO, respectively) with appropriate tdtomato-expressing reporter strains. Serial sectioning, confocal and superresolution microscopy, as well as light-sheet imaging was used to reveal robust labeling of ascending axons and their terminals in expected and unexpected regions.

**Results:**

This strategy provides unprecedented anatomical detail of ascending spinal tracts anterior to the brainstem, and reveals a previously undescribed decussating tract in the ventral hypothalamus (the spinofugal hypothalamic decussating tract, or shxt). The absence of Hoxb8-suppressing elements led to multiple instances of ectopic reporter expression in Hoxb8-Cre mice (retinal ganglion and vomeronasal axons, anterior thalamic nuclei and their projections to the anterior cingulate and retrosplenial cortices and subiculum, and a population of astrocytes at the cephalic flexure) and Hoxb8-FlpO mice (Cajal–Retzius cells of the dentate gyrus, and mesenchymal cells of the choroid plexus). While targeted transgenic lines were similar in terms of known spinofugal projections, Hoxb8-IRES-Cre reporters had an additional projection to the core of the facial motor nucleus, and more abundant Hoxb8-lineage microglia scattered throughout the brain than Hoxb8-T2A-FlpO (or any other) mice, suggesting dysregulated Hoxb8-driven reporter expression in one or both lines.

**Discussion:**

This work complements structural and connectivity atlases of the mouse central nervous system, and provides a platform upon which their reactions to injury or disease can be studied. Ectopic Hoxb8-driven recombinase expression may also be a useful tool to study structure and function of other cell populations in non-targeted lines.

## 1 Introduction

Animal behaviour relies on the ability to detect and respond to external and internal stimuli. In the somatosensory system, primary afferent neurons of the dorsal root ganglion are the first to be activated upon natural stimulation. This information is transmitted to the spinal cord and brainstem where it is modulated by local and descending influences. Long-distance projection neurons then carry this processed information to supraspinal structures where it can be integrated and acted upon. The positioning of ascending tracts in the mouse spinal cord has been well-established (Watson and Harrison, 2012), but their routes to supraspinal termination zones – particularly in the midbrain and thalamus – is less well-understood, despite a relatively deep knowledge of their cells of origin in the spinal cord (Kayalioglu, 2009).

Historically, the primary method to label axons ascending from the spinal cord to the brain (“spinofugal” projections) – a prerequisite for investigating the circuitry responsible for relaying sensory information – has employed degeneration studies and anterograde and retrograde tracing techniques. There are several limitations associated with these approaches (Cliffer et al., 1991; Saleeba et al., 2019), the most pertinent of which when it comes to the spinal cord is the relatively small number of neurons that anterograde tracers can reliably label, especially when the neurons of interest are as widely distributed as they are along the cord’s length. This prompted us to ask whether genes expressed specifically at the level of the spinal cord during development could be exploited as drivers for reporter expression to permanently label spinofugal projections.

Given their restricted expression to the caudal neuraxis (brainstem and spinal cord), Hox genes represent promising candidates. Rostral expression boundaries of the different Hox proteins are correlated with their position in their respective clusters (i.e., 3′-positioned Hox genes expressed more rostrally, 5′-positioned Hox genes expressed more caudally, aka spatial colinearity). One family of Hox genes (Hoxb) shows a marked restriction in expression to the dorsal (alar) plate of the developing spinal cord (Graham et al., 1991), implicating a role in the establishment of spinofugal pathways. Indeed, in mice lacking Hoxb8, fewer neurons of the dorsal spinal cord are generated, their usual laminar positioning is disturbed, and their afferent input is disorganized; these are accompanied by sensory deficits including hyposensitivity to heat and abnormal itch (Holstege et al., 2008), although the latter phenomenon has been attributed to the absence of microglial Hoxb8 expression (Chen et al., 2010).

These findings point to Hoxb8 as a useful tool to drive expression of reporter molecules to label spinofugal axons. We have evaluated the utility of lineage tracing in four transgenic mouse lines (Hoxb8-Cre, Hoxb8-FlpO, Hoxb8-T2A-FlpO and Hoxb8-IRES-Cre) for the bulk-labeling of all spinofugal projections.

The earliest efforts to generate Hoxb8 reporter constructs were carried out by the Deschamps group, who aimed to identify the regulatory elements responsible for setting the anterior expression boundary of Hoxb8 in the brainstem (Charité et al., 1995). An 11 kb segment (located upstream of the Hoxb8 gene and including part of the adjacent Hoxb9 sequence) was used to generate one line of LacZ reporter mice, and partially mimicked endogenous Hoxb8 expression but with a more caudal expression boundary (the fourth cervical spinal segment rather than the brainstem). Two of the transgenic mouse lines used in this paper were generated with this DNA sequence fused either to a Cre (Witschi et al., 2010) or a FlpO recombinase cassette (Zhang et al., 2018).

We also use two mouse lines in which recombinase-encoding sequences were targeted to the endogenous Hoxb8 locus. The first is a knock-in that uses an internal ribosome entry site (IRES) upstream of Cre and should remain under the control of endogenous Hoxb8 regulators (Chen et al., 2010). The second Hoxb8 knock-in drives FlpO recombinase expression, but in this case a sequence encoding the self-cleaving peptide T2A was inserted before the Hoxb8 stop codon in exon 2 and was fused to FlpO before the 3’ UTR (Bohic et al., 2023). We have recently partially characterized this mouse line (Bohic et al., 2023).

Here, we anatomically characterize these four different Hoxb8 reporter lines using confocal, suprerresolution, and light-sheet microscopy. All models demonstrate bulk-labeling of ascending projections to the brain, but with important differences given rostral expression boundaries in targeted and non-targeted transgenic mice. The routes taken by these axons through the pons, midbrain and thalamus have also been clarified, and we describe a new decussating tract of spinofugal axons ventral to the hypothalamus. Additionally, the various transgenic mice differ in terms of reporter-positive cell populations, pointing towards altered regulatory control of each transgene with respect to that of endogenous Hoxb8.

## 2 Materials and methods

### 2.1 Animals and specimen collection

**Figure 1:**
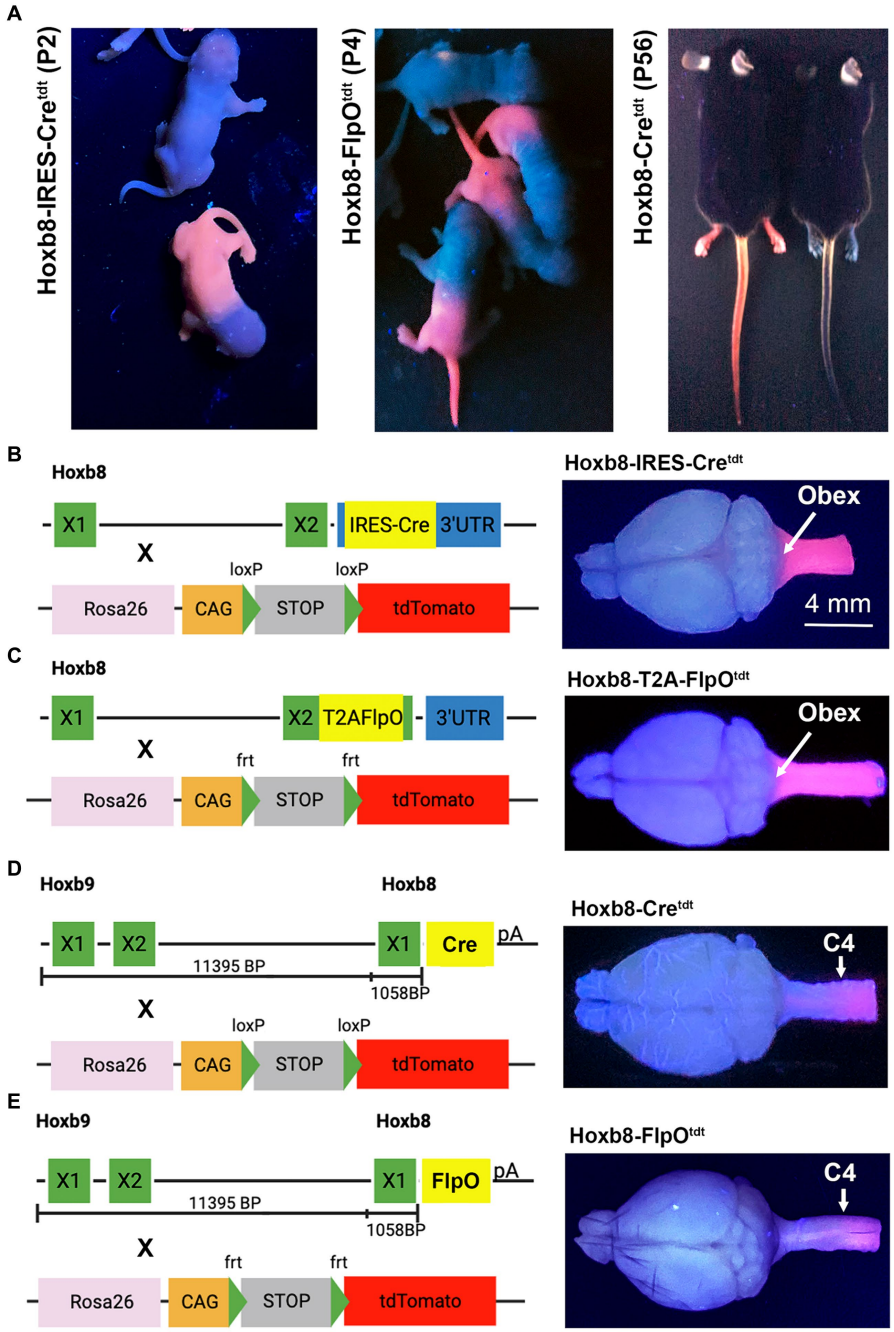
Hoxb8 reporter phenotypes and genotypes. **(A)** tdtomato fluorescence of mouse pups and adult mice under ultraviolet illumination. Note more caudal reporter boundary (sparing forelimbs) in non-targeted transgenic pups (Hoxb8-Cre^tdt^). Non-fluorescent mice in each panel are littermates from crosses between mice hemizygous for the recombinase and homozygous for tdtomato. “X” indicates exons. **(B–E)** Hoxb8-driven recombinase constructs, reporter strains, and ultraviolet-illuminated brains for targeted **(B,C)** and non-targeted **(D,E)** transgenic mice.

All experiments were carried out in accordance with guidelines of the Canadian Council on Animal Care and the UBC Animal Care Committee. Hoxb8-IRES-Cre (RRID:IMSR_JAX:035978), Hoxb8-Cre (obtained from the lab of Hanns Zeilhofer, ETH Zurich, Switzerland), Hoxb8-FlpO (C57BL/6-Tg(Hoxb8-flpo)11Oki/Kctt, sperm obtained from the European Mutant Mouse Archive) mice were crossed with appropriate TdTomato (tdt) reporter mice (Ai14, 007914, RRID: IMSR_JAX:007914; Ai65, 021875, RRDI: IMSR_JAX:021875) (Figures 1A–E). Mice were kept on a standard diet and light cycle.

One male and one female (*n* = 2) of the Hoxb8-IRES-Cre^tdt^ strain, two males and one female (*n* = 3) of the Hoxb8-Cre^tdt^ strain, and three males and one female (*n* = 4) of the Hoxb8-FlpO^tdt^ strain 8–12 weeks of age were deeply anesthetized with a 1,500 mg/kg intraperitoneal injection of urethane and subsequently perfused with heparinized saline followed by 4% paraformaldehyde (PF) in 0.1 M phosphate buffer (PB). The brains and rostral portion of the spinal cords were removed and fixed overnight in 4% PF in 0.1 MPB, after which they were equilibrated in 20% sucrose in 0.1 M PB. Four percent PF-fixed brains and cervical spinal cords of Hoxb8-T2A-FlpO^tdt^ (*n* = 2, 1 M, 1F) mice were generously provided by Dr. Victoria Abraria (Rutgers University) (Bohic et al., 2023). Photos of each specimen were obtained under UV illumination visualize the rostral boundary of reporter expression in the brain and cervical cord. The brains were then frozen in optical cutting temperature compound and stored at −80°C.

One neonatal Hoxb8-FlpO^tdt^ mouse pup (2d) was deeply anesthetized using isoflurane and decapitated. The head was fixed for 48 h in 4% PF in 0.1 M PB, after which the brain was removed, fixed, and equilibrated in sucrose as described above.

### 2.2 Lateral spinal hemisection

Four additional Hoxb8-Cre^tdt^ mice underwent lateral hemisection at C3 in order to injure all reporter-positive spinofugal axons on one side of the animal. Immediately prior to surgery, animals received subcutaneous injections of buprenorphine (0.01 mg/kg) and lactated ringers (0.1 mL). The animals were anesthetized with isoflurane (5% induction, 2–3% maintenance) following which eye lubricant was liberally applied, the upper back was shaved (excess hair removed with depilatory cream), and the skin surface was disinfected with ethanol and chlorhexidine. Mice received a midline incision over the upper cervical spinal cord, and a C3-C4 laminectomy was performed. Hemisections were made by first piercing the dura and cord with a 25 gauge needle just to the right of the dorsal midline vein. One blade of a pair of microscissors was then inserted into the puncture to the bottom of the spinal canal; the other was placed outside the lateral side of the left hemicord and the scissors were closed. The muscle and skin layers were closed with sutures. Mice survived for 6 days (*n* = 1), 2 weeks (*n* = 2) or 4 weeks (*n* = 1) during which they underwent appropriate monitoring and post-surgical care. Mice received buprenorphine (0.01 mg/kg) and lactated ringers (0.1 mL) subcutaneously three times daily for the first 3 days post-surgery.

### 2.3 Tissue processing

Eight brains from intact mice (two adults of each genotype) were serially-sectioned in the coronal plane at 100 microns using a Cryostat (Micron). One male Hoxb8-Cre^tdt^ and one male Hoxb8-FlpO^tdt^ brain were also sectioned parasagittally at 100 microns. An additional male Hoxb8-FlpO^tdt^ mouse was sectioned coronally at 50 micron sections and thaw mounted to Superfrost Plus slides. The 2d-old mouse pup brain was also sectioned coronally at 100 microns. Four-mil (~101 mm) rectangular adhesive vinyl frames custom made on a Cricut (Explore Air) were applied to Superfrost Plus slides before thaw-mounting sections to prevent tissue compression during coverslipping. The slides were air dried for a minimum of 2 h and washed with phosphate-buffered saline (PBS, 2x, 10 min each). Slides were then delipidated overnight with Cubic-L (TCI, Cat# T3740) to which NaCl was added (to 0.45%) to prevent tissue swelling and detachment from the slides. The following day, the refractive index (RI = 1.520) was matched to glass using Cubic-R+(M) (TCI, Cat# T3741) in 2 × 20 minute applications. Finally, slides were coverslipped using 24 × 50 mm coverslips and Cubic mounting medium (TCI, Cat# M3294) or ProLong Glass mountant (ThermoFisher Cat# P36984).

### 2.4 Immunohistochemistry

Sections were permeabilized and blocked with 10% normal donkey serum and 0.2% Triton X-100 plus 0.02% sodium azide in PBS (PBSTx azide) for 1 hour before the primary antibodies were applied (Table 1) for ~48 h. Prior to application of the secondary antibodies (Table 1), the slides were washed three times (5 min each) with PBS. Secondary antibodies were applied overnight at room temperature and subsequently washed 3 times for 5 min each in PBS. The refractive index was matched to glass using Cubic-R+(M) (TCI, Cat#T3741) in one 20-min application. Slides were coverslipped using 24x50mm coverslips and the Cubic mounting media (TCI, Cat# M3294) before being sealed with nail varnish. Slides with 50 micron-thick sections were not cleared with Cubic reagents, and instead coverslipped using ProLong Glass anti-fade mounting media (Invitrogen, P36984).

**Table 1:** Antibodies for immunohistochemistry.

Antigen	Source	Conjugate/Dilution
Sox9	(R and D Systems Cat# AF3075, RRID:AB_2194160)	NA/1:200
NeuN (Fox3)	Millipore Cat# MAB377, RRID:AB_2298772	NA/1:100
p73	Abcam Cat# ab40658, RRID:AB_776999	NA/1:200
Iba1	FUJIFILM Wako Shibayagi Cat# 019–19,741, RRID:AB_839504	NA/1:1000
Iba1	Cell Signaling Technology, Cat#17198S, RRID: AB_2820254	Alexa 488/1:250
PDGFRa	R and D Systems Cat# AF1062, RRID:AB_2236897	NA/1:200
Goat IgG	Jackson ImmunoResearch, Cat#705–165-147, RRID: AB_2307351	Alexa 488/1:500
Mouse IgG	Jackson ImmunoResearch, Cat# 715–546-151 RRID: AB_2340850	Alexa 488/1:500
Mouse IgG	Jackson ImmunoResearch, Cat#715–605-150, RRID: AB_2340862	Alexa 647/1:500
Rabbit IgG	Abcam, Cat#ab96894	Dy 650/1:500

### 2.5 Nissl staining—spinal cords

The spinal cords (cervical to lumbar) were harvested from an additional five PF-perfused mice (one of each genotype and a second Hoxb8-Cre^tdt^ mouse) to examine neuronal recombination in the spinal cord. In this case, 50-micron frozen sections were thaw-mounted onto glass slides and counterstained with NeuroTrace^™^ 640/660 Deep-Red Fluorescent Nissl Stain (1:200 for 2 h, ThermoFisher Catalog number: N21483).

### 2.6 Image acquisition

Images of coronally-sectioned adult brains were taken using a 5x objective on a Zeiss LSM800 scanning confocal microscope and Zen Blue software. Each brain section was imaged in all dimensions using tiling and z-stacking (4–6 z-slices with an optical section thickness of 40–50 microns after orthogonal projections). The images were stitched during acquisition, orthogonally projected, and a single pixel filter was applied to remove digital gain artifacts. To begin registration to the Allen Mouse Atlas coronal average template, any rotation of the microscope images in the rostro-caudal plane was determined using QuPath. The Allen Mouse Atlas average coronal template was then digitally sectioned to account for this rotation (which was always less than ±2.5^o^). Fiducials were selected that included areas of high TdTomato reporter intensity, major surface structures, and internal landmarks (ex: inner surfaces of the ventricles). In FIJI, 8–12 fiducials were selected on each Allen Mouse Atlas section and then the same landmarks were chosen on the corresponding microscope image (FIJI > region of interest). The thickness of the microscope images and the Allen Mouse Atlas slices were not identical, therefore adjustments were made by skipping an atlas slice or doubling up (i.e., registering multiple microscope images to the same Allen Mouse Atlas slice) to minimize divergence. In general, an effort was made to keep the fiducials in the same location and only make gradual changes as we moved through the Allen mouse Atlas along the z-axis. The images were registered to the Allen Mouse Atlas coronal average template using FIJI (moving least squares with similarity method in the registration plugin) and stacked (in tools, stack sorter). It should be noted that the slight rotation along the rostro-caudal axis of our sections (and registration to a slightly rotated average coronal template).

Images of sections for immunohistochemistry were taken using a 20x or 63x objective using a Zeiss LSM800 scanning confocal microscope and Zen Blue software for the above sections. Super-resolution microscopy (63x) was used to capture images of p73-labeled cells (Airyscan).

### 2.7 Light sheet microscopy

One intact Hoxb8-IRES-Cre^tdt^ mouse and one Hoxb8-Cre^tdt^ mouse, hemisected 28 days earlier, were deeply anesthetized with a 1,500 mg/kg intraperitoneal injection of urethane and perfused with heparinized saline followed by 4% PF in 0.1 M PB. Their brains were removed and fixed overnight in 4% PF in 0.1 M PB. Brains were washed for 6 h (3 × 2 h) in PBS on a room temperature shaker protected from light. Brains were delipidated for 7 days with Cubic-L (TCI, Cat #T3740) to which NaCl was added (to 0.45%) to prevent tissue swelling in a 37°C incubated shaker protected from light. The Cubic-L solution was changed daily for the first 4 days, after which it was changed every second day. The refractive index (RI = 1.520) was matched to glass using Cubic-R+(N) (TCI, Cat# T3983) for 2 days on a room temperature shaker protected from light. The cleared cerebella were embedded in 2.5% agarose in Cubic-R+(N) (TCI, Cat#T3983). Images of the cerebella were acquired using a Zeiss Z.1 Light sheet microscope with Zen Black software with tiling and z-stacking.

## 3 Results

### 3.1 Gross tdtomato expression in Hoxb8 reporter mice

Transgenic Hoxb8-driven reporter expression was easily identified with UV illumination (Figure 1A). Fluorescence in knock-in strains extended from the tail to the nape of the neck, and included the forelimb; this was not the case in non-targeted strains, in which the forelimb was spared, and reporter expression was limited to skin below mid-torso (Figure 1A). Developmental Hoxb8 mRNA expression extends from the caudal end of the spinal cord to the brainstem (Witschi et al., 2010). This is reflected in the extent of reporter expression in the CNS of Hoxb8-IRES-Cre^tdt^ and Hoxb8-T2A-FlpO^tdt^ knock-in strains (Bohic et al., 2023 and Figures 1B–E), as demonstrated under UV illumination. In the transgenic reporter models (Hoxb8-Cre^tdt^ and Hoxb8-FlpO^tdt^), the rostral expression boundary was close to C4 (Figures 1D,E), as expected based on previously demonstrated expression of the transgenic constructs in mouse embryos and adult reporters (Charité et al., 1995; Witschi et al., 2010). In three of the mouse lines maintained in our facility, recombination occasionally occurred throughout the body. Such mice were excluded from analyses.

### 3.2 Reporter expression in the spinal cord

**Figure 2:**
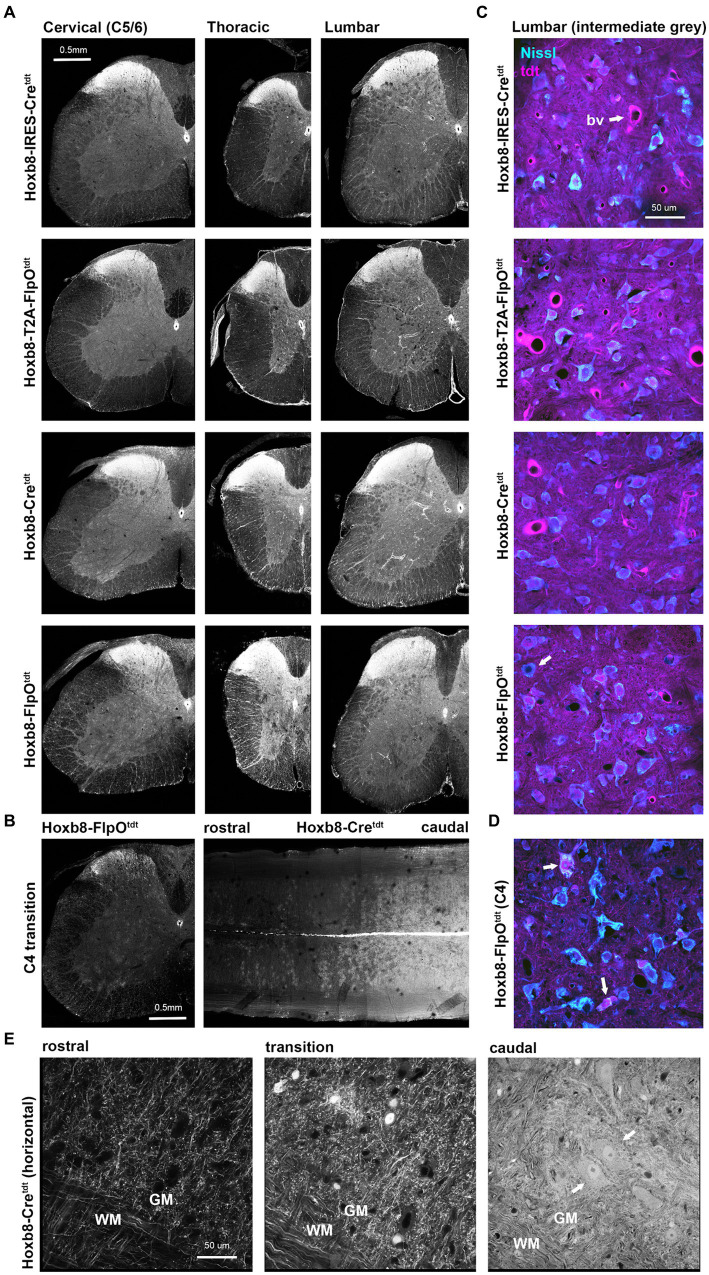
Reporter distribution in the spinal cords of Hoxb8 reporter mice. **(A)** Spinal cord cross sections from cervical (below C4), thoracic and lumbar cords of all reporter strains. **(B)** Cross section and equatorial horizontal sections at the Hoxb8 transition zone (C4) from non-targeted transgenic strains. **(C)** Higher-power images of Nissl-stained sections of the intermediate lumbar grey matter illustrating near-complete recombination in neurons. A rare example of a reporter negative neuron is indicated in the bottom panel (arrow). bv, blood vessel. **(D)** As in **(C)** but at the Hoxb8 transition zone; recombined neurons are indicated with arrows. **(E)** horizontal sections from the ventral cord of Hoxb8-Cre^tdt^ mice spanning the hoxb8-transition zone. Recombined motoneurons are indicated by arrows. WM, white matter; GM, grey matter.

With rare exceptions, all spinal cord neurons caudal to Hoxb8 expression boundaries had undergone recombination in all reporter lines (Figure 2). Tdtomato intensity was expectedly intense in the dorsal grey matter given the high neuronal density. In non-targeted transgenics, only axon terminals could be detected in the cord above C4 (Figure 2E). In the thoracic and lumbar cords (but not the cervical cord), vascular-associated cells were apparent, in line with the mesodermal distribution of Hoxb8 expression during development (Deschamps et al., 1999). We are thus able to conclude that all spinofugal projections are tdtomato-positive (but only up to C4 in non-targeted reporter strains).

### 3.3 Spinofugal projections in Hoxb8-reporter mice

**Figure 3:**
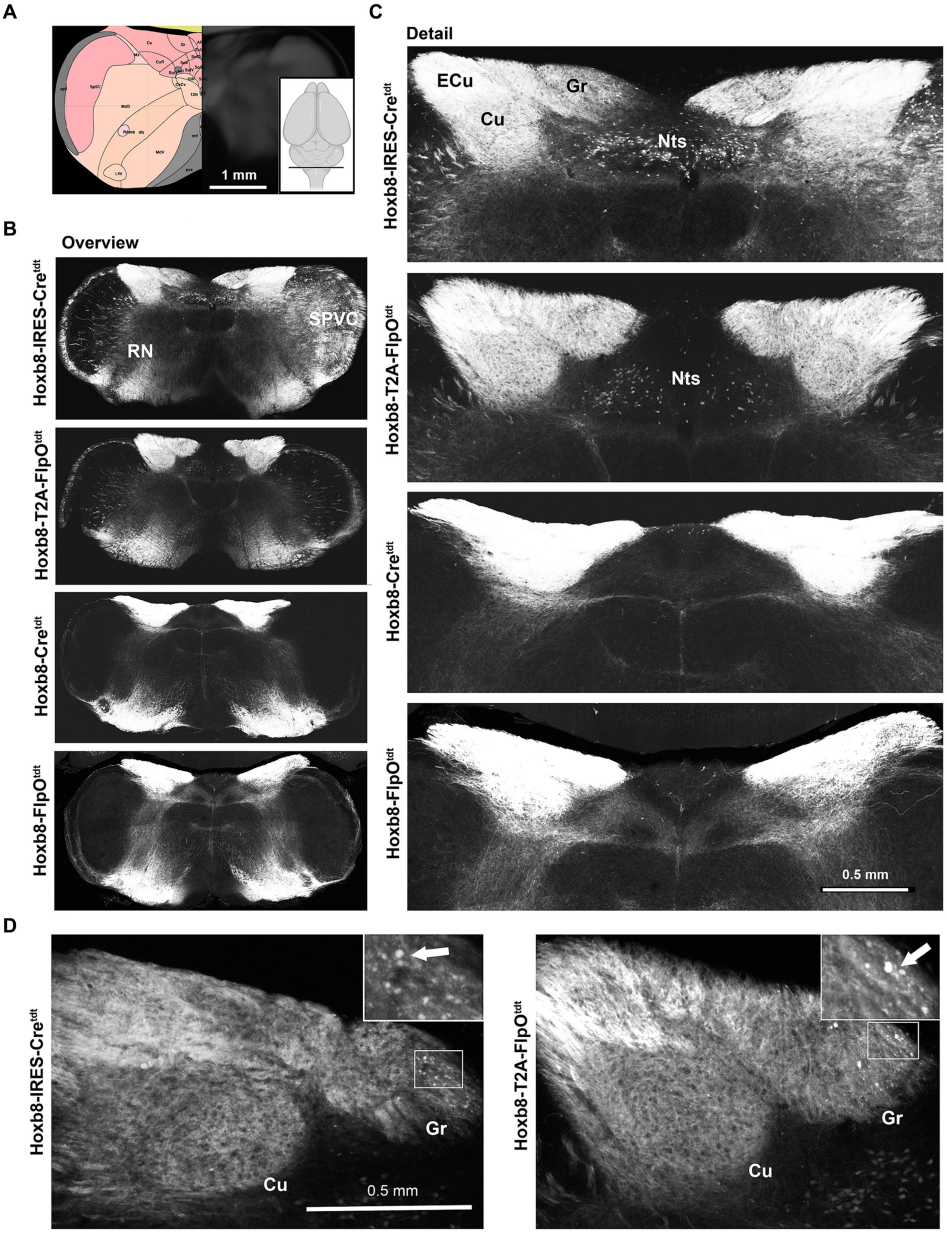
Medullary reporter fluorescence in Hoxb8 reporter strains. **(A)** Schematic indicating plane of section (inset) and the equivalent image from the YSK Unified Anatomical Atlas (https://kimlab.io/brain-map/atlas/). **(B)** Overviews of reporter fluorescence from each strain. **(C)** Detail of the dorsal medulla. Reporter-positive neurons are present within the nucleus of the solitary tract (Nts) of targeted strains, and absent from the others. **(D)** Reporter-positive neurons (arrows) in the gracile nucleus of targeted transgenic reporter strains.

**Figure 4:**
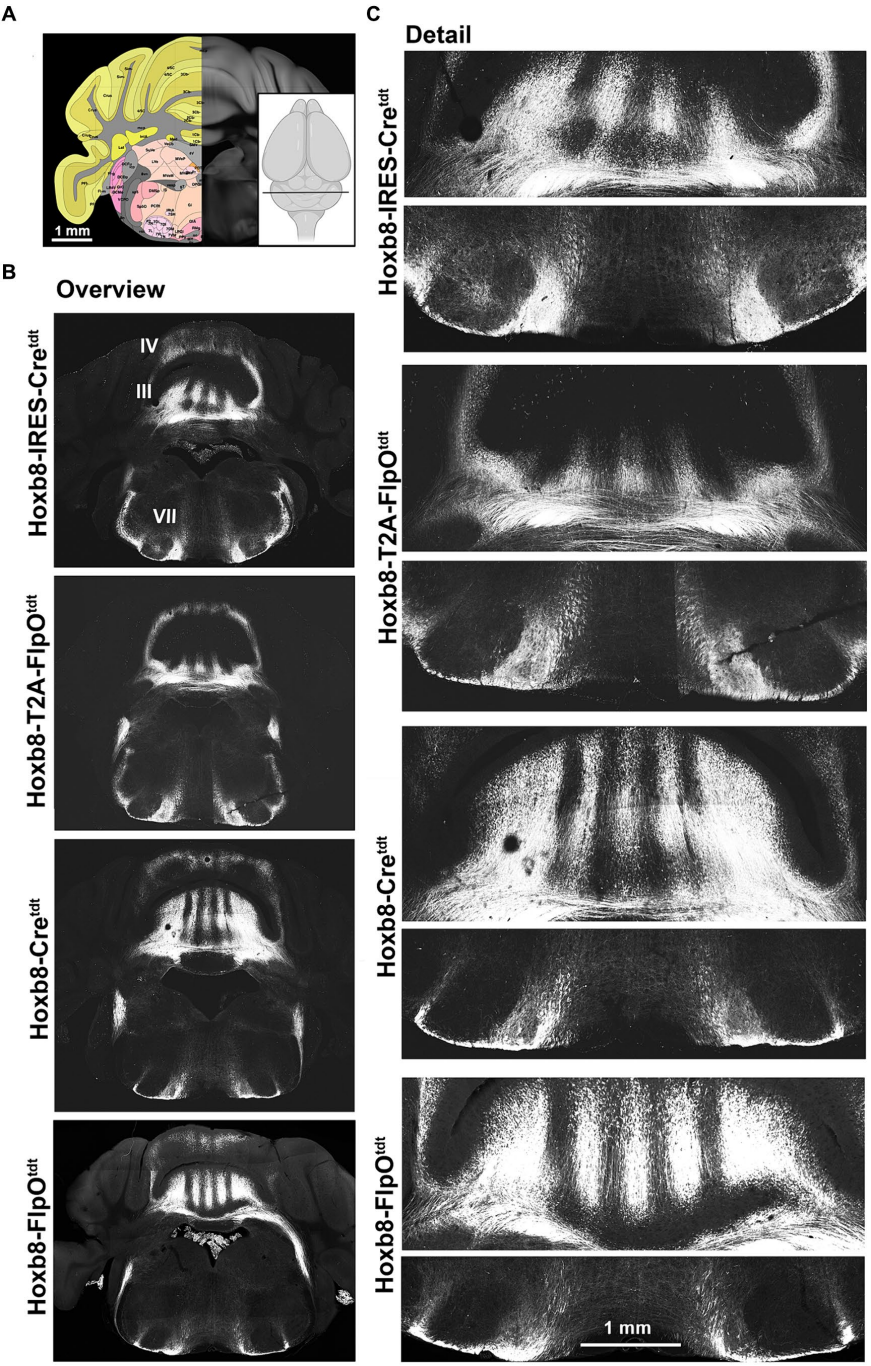
Hindbrain reporter fluorescence in Hoxb8 reporter strains. **(A)** Schematic indicating plane of section (inset) and the equivalent image from the YSK Unified Anatomical Atlas (https://kimlab.io/brain-map/atlas/). **(B)** Overviews of reporter fluorescence from each strain (III and IV indicate cerebellar lobules; VII indicates the facial motor nucleus). **(C)** Details of the cerebellum (lobule III) and ventral hindbrain.

**Figure 5:**
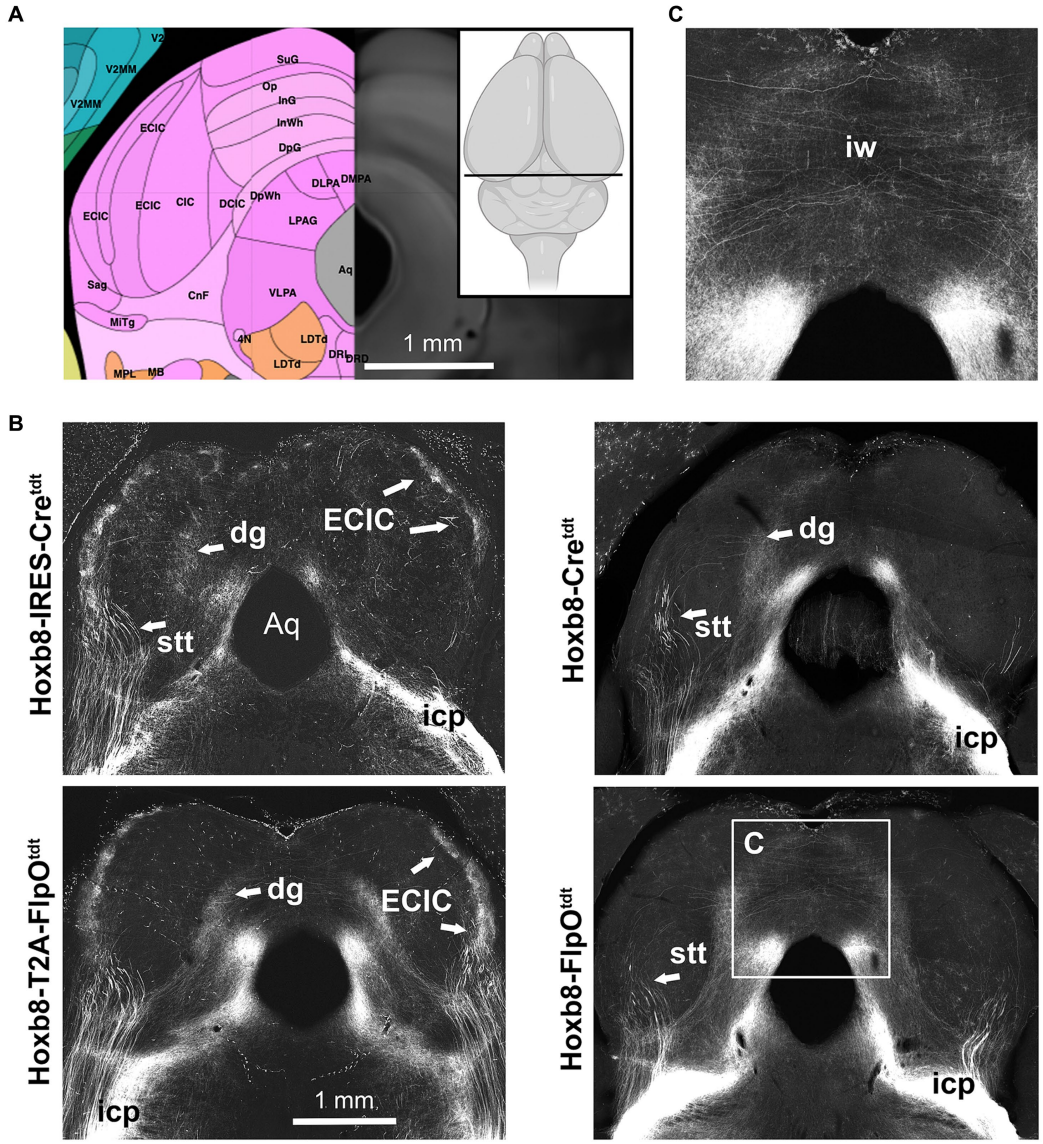
Midbrain reporter fluorescence in Hoxb8 reporter strains: inferior colliculus. **(A)** Schematic indicating plane of section (inset) and the equivalent image from the YSK Unified Anatomical Atlas (https://kimlab.io/brain-map/atlas/). **(B)** Reporter fluorescence from each strain. Arrows indicate the external cortex of the inferior colliculus (ECIC), absent from non-targeted transgenic strains. **(C)** Higher-power view of decussating axons in the intermediate white matter of the superior colliculus (iw). Aq, cerebral aqueduct.

**Figure 6:**
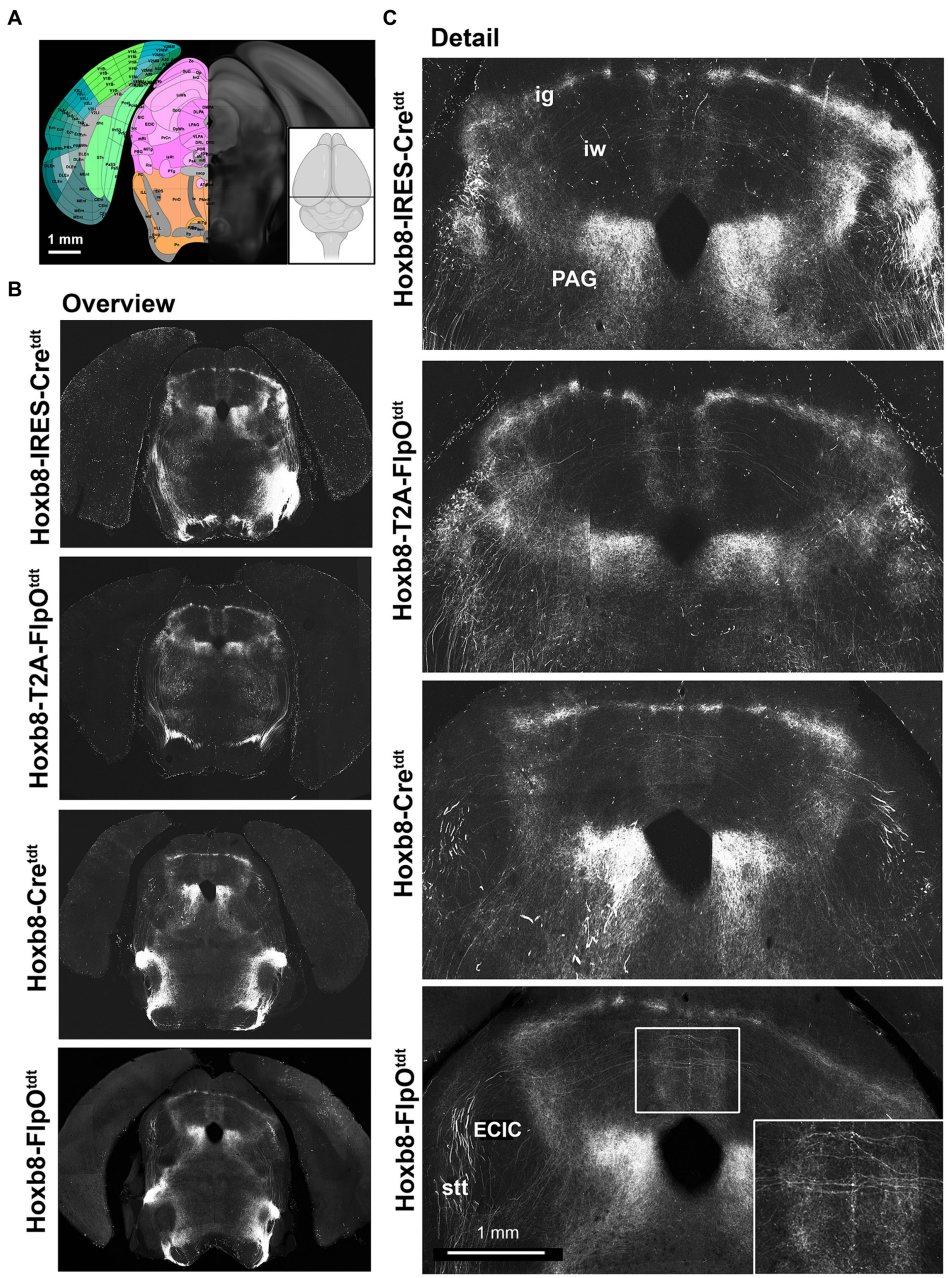
Midbrain reporter fluorescence in Hoxb8 reporter strains: superior colliculus. **(A)** Schematic indicating plane of section (inset) and the equivalent image from the YSK Unified Anatomical Atlas (https://kimlab.io/brain-map/atlas/). **(B)** Overviews of reporter fluorescence from each strain. **(C)** Details of the dorsal midbrain. Ig, intermediate grey layer of the superior colliculus; iw, intermediate white layer of the superior colliculus; PAG, periaqueductal grey; ECIC, external nucleus of the inferior colliculus; stt, spinothalamic tract.

**Figure 7:**
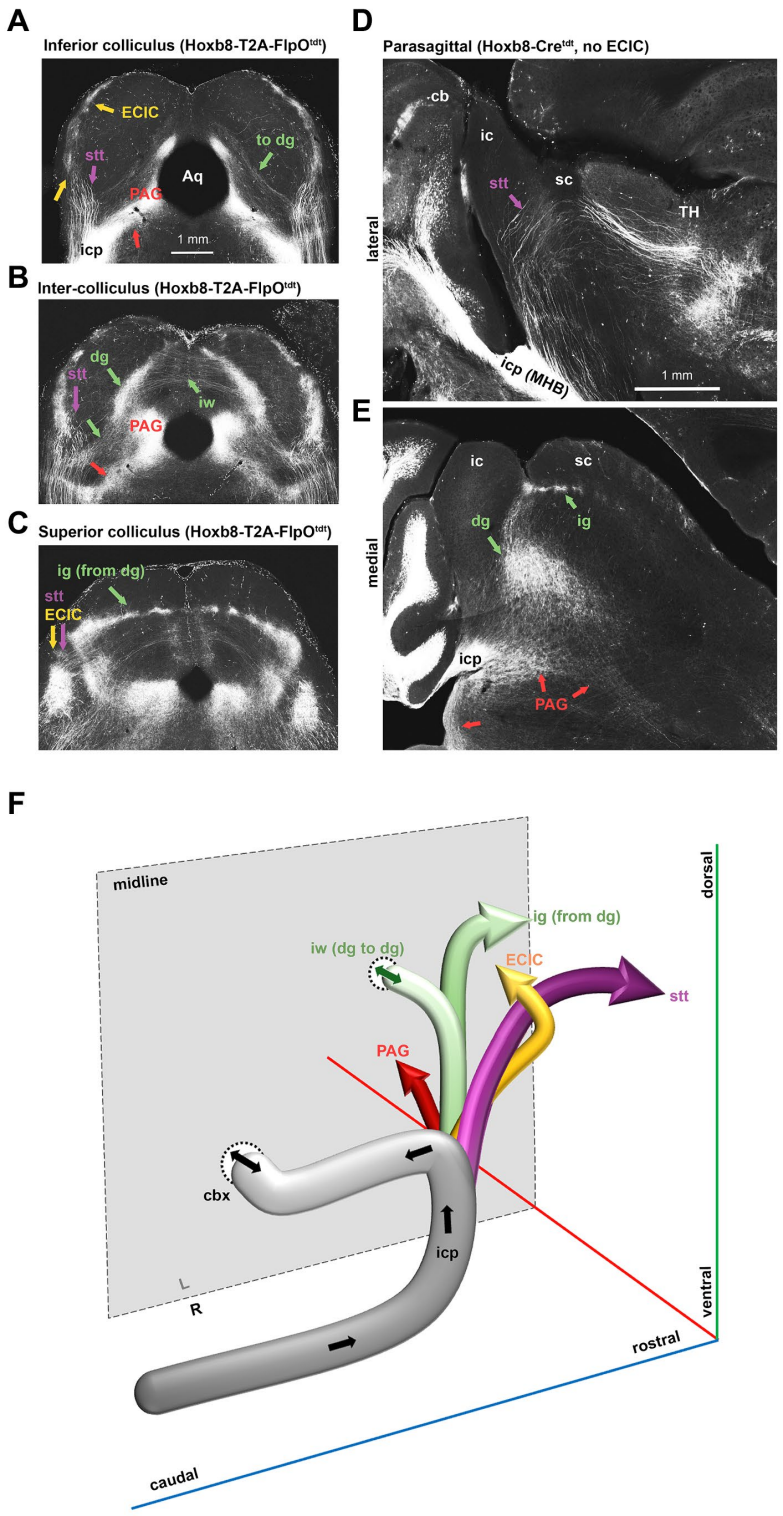
Dorsal spinofugal pathways to the midbrain and thalamus. **(A–C)** Collicular transition from inferior **(A)** to superior in a Hoxb8-T2A- FlpOtdt mouse. **(D,E)** Parasagittal sections from a Hoxb8-Cretdt mouse illustrating the origins of the dorsally-coursing spinocerebellar tract from the inferior cerebellar peduncle (icp) at the midbrain-hindbrain boundary (MHB) (D) and the continuity of projections from the deep grey (dg) to the intermediate grey (ig) **(E)**. **(F)** Schematic of four separate projections to the midbrain and thalamus emerging from the icp. cb, cerebellum; cbx, cerebellar commissure, dg, deep grey matter of the superior colliculus; ECIC, external nucleus of the inferior colliculus; ig, intermediate grey of the superior colliculus; iw, intermediate white matter of the superior colliculus; PAG, periaqueductal grey; Stt, spinothalamic tract; TH, thalamus.

**Figure 8:**
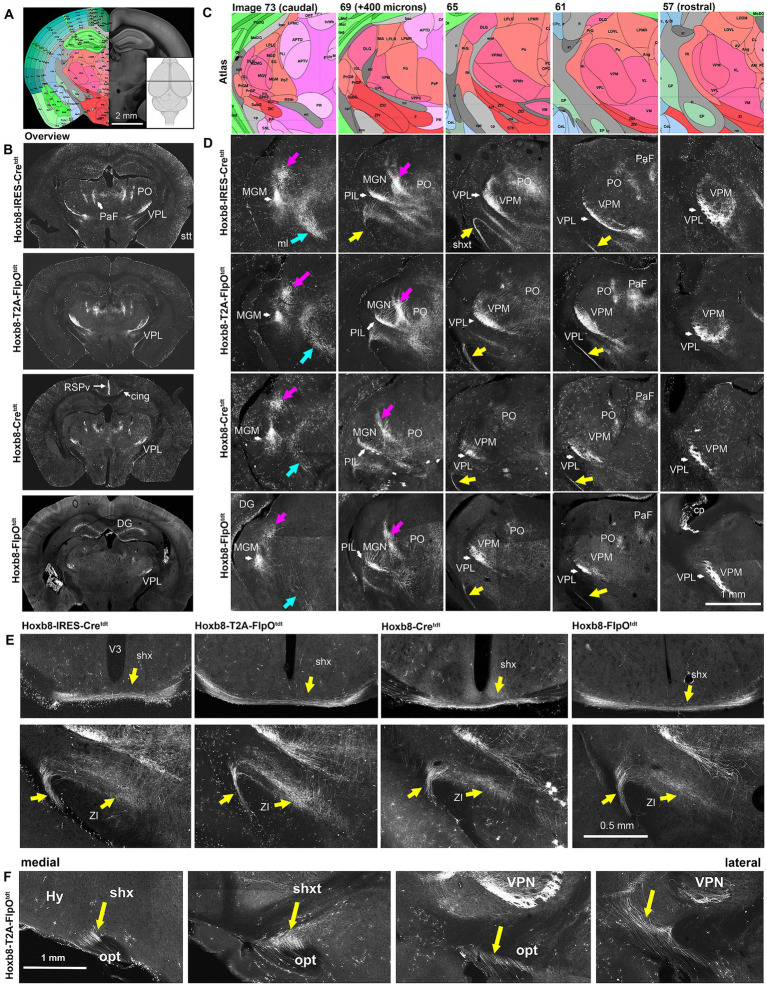
Reporter fluorescence in the thalamus of Hoxb8 reporter strains. **(A)**, Schematic indicating plane of section (inset) and the equivalent image from the YSK Unified Anatomical Atlas (https://kimlab.io/brain-map/atlas/). **(B)** Overviews of reporter fluorescence from each strain. **(C,D)** Higher-power schematics (reference atlas image numbers indicated) and photomicrographs of the posterior thalamus at 400 micron intervals. Magenta arrows indicate spinothalamic tract (stt) axons; cyan arrows indicate the medial lemniscus (ml); yellow arrows indicate the spinofugal hypothalamic decussating tract (shxt). **(E)** Higher power views of the shx, shxt and a possible termination zone in the zona incerta (ZI) in four Hoxb8 reporter lines. **(F)** Parasagittal sections (medial to lateral) from a Hoxb8-T2A-FlpO^tdt^ mouse of the shxt. Cing, cingulum bundle; cp, choroid plexus; DG, dentate gyrus; MGM, medial part of the medial geniculate nucleus; MGN, medial geniculate nucleus; PaF, parafascicular nucleus; PO, posterior complex of the thalamus; PoT, posterior triangular complex; RSPv, retrosplenial cortex; V3, third ventricle; VPM, ventral posterior medial nucleus of the thalamus; VPL, ventral posterior nucleus of the thalamus.

**Figure 9:**
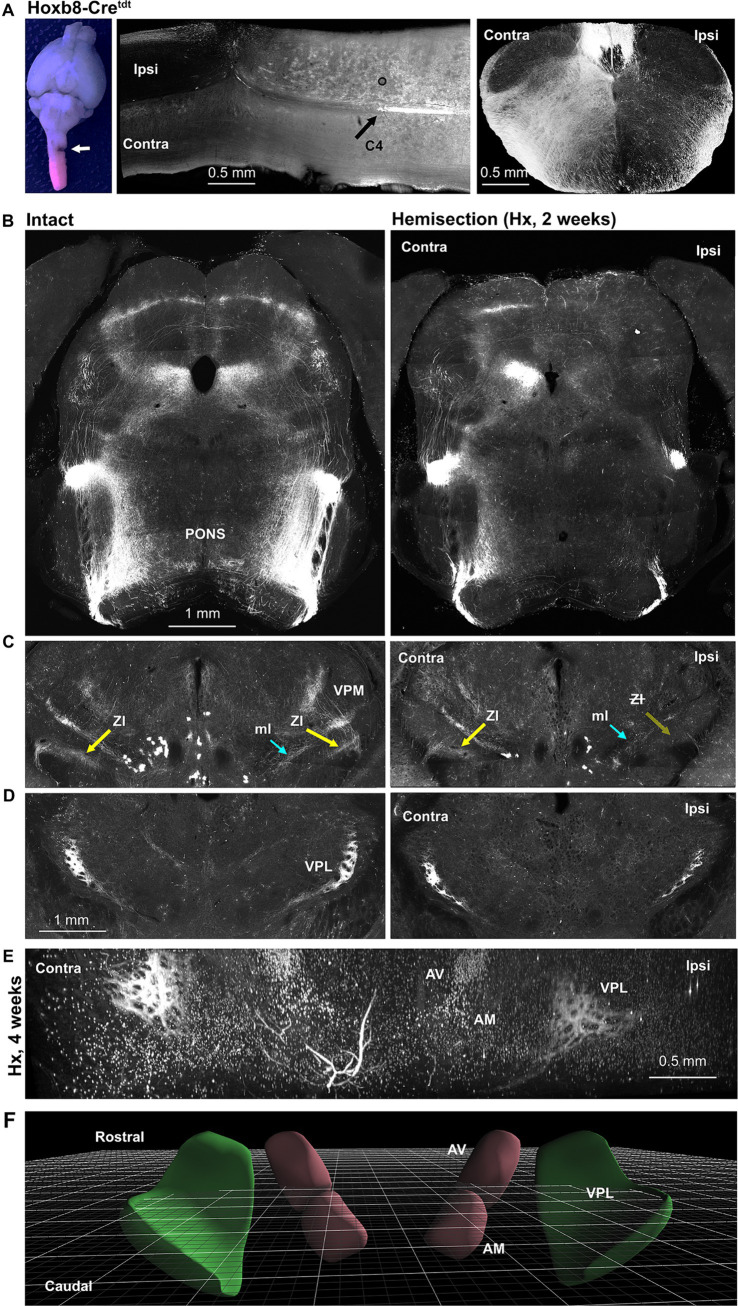
Lateral hemisection reveals origins of spinofugal axons: Hoxb8-Cre^tdt^ mice. **(A)** Gross view of the site of C3 hemisection (left panel, arrow), a horizontal section through the injury site middle panel, and a cross sectional view demonstrating ipsilateral terminal degeneration. **(B)** Loss of spinotetcal and spinoponitne axons ipsilaterally. **(C)** Thalamic projections (Zona incerta, ZI, highlighted) are largely preserved contralateral to injury, implying an ipsilateral pathway decussating in the diencephalon. **(D)** The anterior VPL nucleus is spared contralaterally, and only partly diminished ipsilaterally. **(E)** Light-sheet imaging demonstrates anterior VPL nucleus sparing at 4 weeks post-hemisection (Hx). **(F)** Schematic demonstrating orientation of perspective in E (3D viewer from www.connectivity.brain-map.org). Cyan arrows indicate the medial lemniscus (ml); yellow arrows indicate the spinofugal hypothalamic decussating tract (shxt).

**Figure 10:**
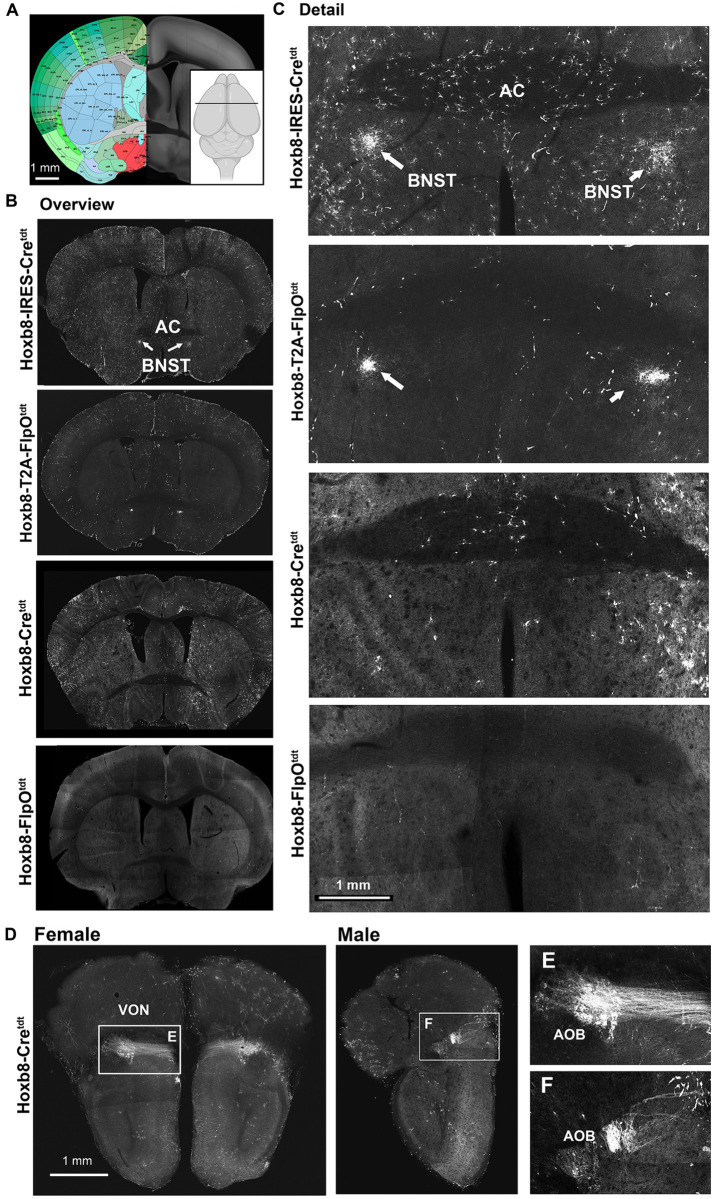
Reporter fluorescence at (and rostral to) the level of the anterior commissure (AC) in Hoxb8 reporter strains. **(A)** Schematic indicating plane of section (inset) and the equivalent image from the YSK Unified Anatomical Atlas (https://kimlab.io/brain-map/atlas/). **(B)** Overviews of reporter fluorescence from each strain. **(C)** Details of the region surrounding the AC. **(D)** Projections of the vomeronasal nerve (VON) to the accessory olfactory bulb (AOB) in female and male mice. **(E,F)** Details of **(D)**.

Spinofugal projections were labeled in all four Hoxb8 reporter lines, but to slightly different extents depending on brain region (Figures 3–10). Primary afferent terminals (and possibly post-synaptic dorsal column axon terminals) were dense in the gracile (Gr), cuneate (Cu) and external cuneate nuclei (ECu) in all strains (Figures 3B,C), as were a few neuronal somata in the gracile nucleus (but not the other dorsal column nuclei) in targeted transgenics [Figure 3D and (Bohic et al., 2023)]. Other axons extended from ventral ascending tracts into the reticular nuclei (RN). Some neurons were apparent unilaterally in the caudal spinal nucleus of the trigeminal nerve (SPVC) in Hoxb8-IRES-Cre^tdt^ mice due to a slightly yawed section (Figure 3B and data not shown). Labeling was also more pronounced in the spinal tract of the trigeminal nerve in the knock-in models, but these were not trigeminal in origin, given the lack of terminal labeling in the SPVC. Some neurons of the nucleus of the solitary tract were apparent in knock-in strains (NTS, Hoxb8-IRES-Cre^tdt^ > Hoxb8-T2A-FlpO^tdt^); these were absent in the untargeted transgenic reporter mice (Figures 3B,C).

At the level of the anterior cerebellum and facial nucleus (Figure 4), spinocerebellar axons were apparent in lobules as stripes (Figures 4B,C). This was true for lobules II, III, IV, VIII and IX, with no obvious differences in density between strains. In Hoxb8-IRES-Cre^tdt^ mice, but in no other strain, a population of spinofugal (or possibly bulbofugal) axons was present in the core of the facial nucleus (Figure 4C).

In the midbrain (Figures 5, 6), axon terminals of the periaqueductal grey received a dense projection of reporter-positive axons, which extended well into the thalamus. In targeted transgenic models (Hoxb8-IRES-Cre^tdt^ and Hoxb8-T2A-Cre^tdt^), many terminals were apparent in the external nucleus of the inferior colliculus (ECIC), a region that receives input from the spinal cord and brainstem (Aitkin et al., 1978; Davidson et al., 2010). Their absence in non-targeted strains revealed thick axons that continued rostrally (see also Figure 7) toward the thalamus (Figure 7). Axons projecting to the intermediate grey of the superior colliculus formed a meshwork in all mice (Supplementary Video S1). Decussating axons were also present in deeper white matter (intermediate white, iw) of the superior colliculus; these connected the left and right deep grey of the superior colliculus, which itself was continuous with the ig (Figure 7).

The lineage-tracing apporach offered an unprecedented view of the routes taken by spinofugal axons toward the tectum and thalamus (Figure 7). A major point of diversion of anterolateral ascending tracts was at the level of the midbrain-hindbrain boundary, where spinotectal and spinothalamic axons left the inferior cerebellar peduncle, some of which projected to the PAG and the intermediate grey (ig) layer of the superior colliculus (Figure 7). These finer axons with small synaptic terminals additionally projected to two unnamed vertically-oriented bands extending from the ig dorsomedially to the aqueduct ventromedially, and to the lateral border of the PAG ventrolaterally. At this same level, there was a prominent decussation of middling-calibre axons in the intermediate white matter. The thickest axons leaving the middle cerebellar peduncle traveled dorsally in the midbrain, and curved rostrally toward the diencephalon (Figure 7). These, like those in the medial lemniscus, approached the caudal end of the ventral posterior lateral nucleus of the thalamus and the posterior intralaminar nucleus (PIL), but from a dorsal rather than a ventral direction (Figure 7D).

The large-calibre axons ran rostrally through the colliculi toward posterior complex of the thalamus (PO), clustering at the dorso-medial border of the medial geniculate nucleus, and more rostrally at the dorso-medial border of the ventral posterior medial nucleus of the thalamus (VPM) (Figure 8). In the Unified Atlas, this region is labeled as the external medullary lamina rostrally, and is continuous with the superior thalamic radiation (str) caudally. From there, they traversed the MGN and VPM, and arborized in the posterior intralaminar nucleus (PIL) and ventral posterior lateral nucleus (VPL), respectively (Figure 8). The medial part of the medial geniculate nucleus (multimodal part) was particularly heavily labeled in all strains.

The density of dorsal spinothalamic axons was much greater in knock-in strains, probably because the majority of spinothalamic axons originate in the cord above C3 (Davidson et al., 2010). In the pons at the level of the midbrain-hindbrain boundary, a population of axons of the inferior cerebellar peduncle merged with those of the medial lemniscus and ascended toward thalamic nuclei (Figures 7, 8).

A previously undescribed tract of axons emerged from the vicinity of the caudal end of the VPL or possibly the rostral end of the PIL, ran ventrally between the internal capsule and optic tract, and decussated just posterior to the optic chasm (oc) in the ventral-most part of the hypothalamus (Figures 8E,F). We call this the spinofugal hypothalamic decussating tract (shxt), and its decussation (shx). This was more prominent in targeted knock-ins, indicating a contribution from spinal neurons above C4 (Figure 6E). The zona incerta (ZI), appeared to be a site of shxt termination (double yellow arrows in Figure 5A).

While axons in the ventral posterior lateral nucleus (VPL) of the thalamus were apparent in all strains (Figure 8), labeling was slightly less extensive in the mediolateral and rostro-caudal axes in the non-targeted Hoxb8 reporters (Figure 8). Spinofugal axons in the lateral PO were also markedly less prominent in the non-targeted strains. The same was true of putative shxt terminal density in the zona incerta (ZI, Figure 8). More dorsally, labeled axons were apparent in the medial part of the PO, as well as in the parafascicular nucleus (PaF) (Figure 8).

The existence of spinofugal hypothalamic decussation implies that some thalamus-projecting axons either ascend ipsilaterally or contralaterally to their origins in the spinal cord, and then cross (or cross again) in the thalamus. We subjected a small number (4) of Hoxb8-Cre^tdt^ mice to lateral spinal hemisection at C3 (Figure 9) – this manipulation should remove all reporter positive axonal input to the brain on the hemisected side. Gross inspection of tdt fluorescence confirmed loss of reporter-positive terminal ipsilaterally (and to a lesser extent contralaterally) in the cervical cord (Figure 9A), pons and midbrain (Figure 9B), and caudal thalamus including the ZI (Figure 9C). While this indicates ipsilateral ascension from neurons in the uninjured side of the cord, we could not readily trace a path from the ipsilateral thalamus to the contralateral ZI. We did find, however, a surprising preservation of axons in the anterior VPL nucleus (Figure 7D) at two (and even four) weeks post-hemisection (Figures 9E,F), raising the possibility of recurrent projections from the ipsilateral anterior VPL to the more posterior contralateral ZI.

Further rostrally, reporter-positive axons were present in both knock-in strains in a discrete region of the bed nucleus of the stria terminalis (BNST) ventral to the anterior commissure (Figures 10B,C), which we suspect arise from the nucleus of the solitary tract (NTS), based on previous tracing studies in the rat (Riche et al., 1990; Lebow and Chen, 2016), and the absence of recombination in the NTS of non-targeted strains. At the rostral pole of the brain, but only in Hoxb8-Cre^tdt^ mice, axons of the vomeronasal nerve (VON) and their terminations in glomeruli of the accessory olfactory bulb were reporter positive (Figure 10D). Interestingly, these were more prominent in female than male specimens.

### 3.4 Ectopic recombination in non-targeted Hoxb8 transgenic reporter mice

**Figure 11:**
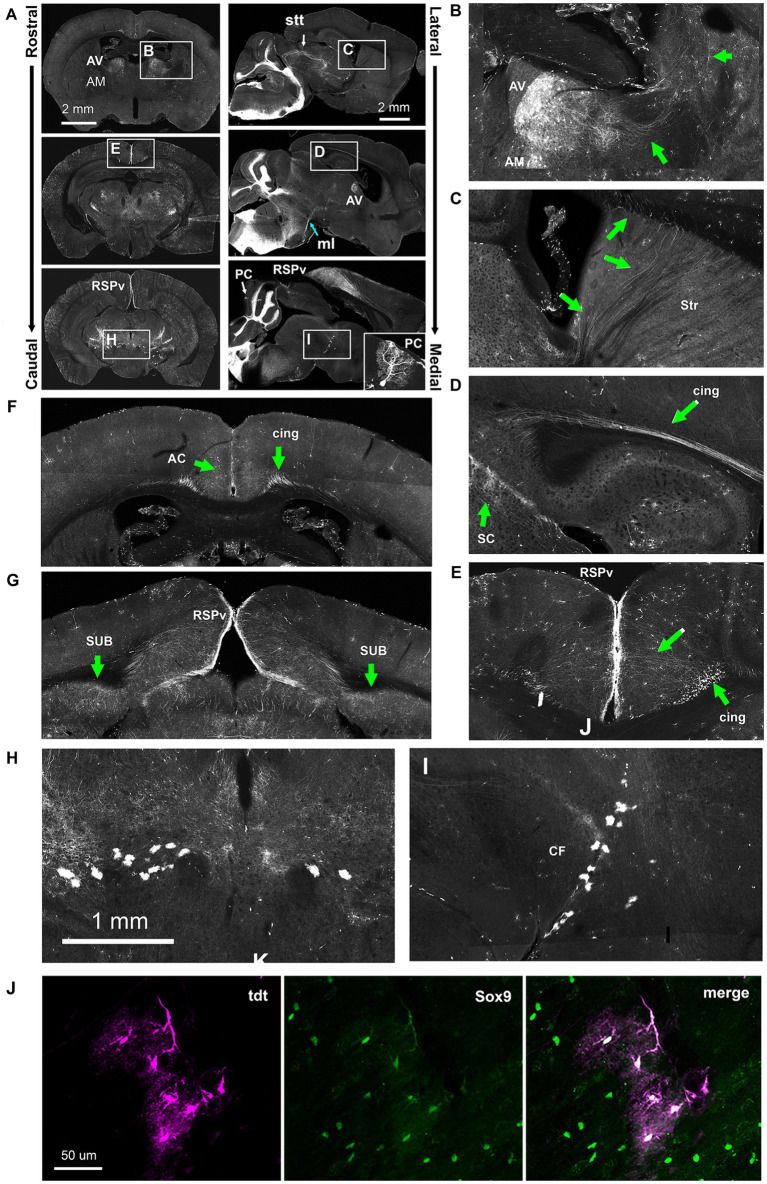
Ectopic Hoxb8-driven reporter expression in Hoxb8-Cre^tdt^ mice. **(A)** Overviews of coronal and sagittal/parasagittal brain sections. Occasional Purkinje cells underwent recombination (PC, inset). **(B)** Detail of the rostral thalamus indicating the anteroventral (AV) and anteromedial (AM) nuclei and the lateral course of axonal projections (green arrows). **(C)** The rostral course of AV and AM-originating axons through the striatum (Str). **(D)** AV and AM axons converge in the cingulum bundle (cing). **(E)** AV and AM-originating axons radiate from the cingulum bundle towards the retrospleinal cortex (RSPv). **(F,G)** Coronal sections from the level of the anterior thalamus **(G)** and the rostral midbrain **(H,I)** showing AV and AM projections to the anterior cingulate cortex (AC), the RSPv, and the subiculum (SUB). **(H,I)** Detail from A showing reporter-positive cells ventral to the cephalic flexure (*CF*). **(J)** Sox9 immunohistochemistry revealed these to be astrocytes. ml, medial lemniscus; stt, spinothalamic tract.

In Hoxb8-Cre^tdt^ mice, scattered neurons in the cortex, hippocampus and cerebellum were reporter-positive, but in no consistent way; a cerebellar Purkinje cell (PC) is shown in the bottom right panel of Figure 11A.

In addition to VON axons (Figure 10), and as reported previously (Szabo et al., 2015), a population of Hoxb8-lineage neurons was present in the anteroventral (AV) and anteromedial (AM) nuclei of the thalamus in the Hoxb8-Cre^tdt^ mice (Figure 11A). We were able to follow the course of their axons, at least from the AV nucleus, laterally (Figure 11B) and anteriorly (Figure 11C) through the dorsal part of the striatum, after which they converged in the cingulum bundle (cing, Figure 11D). In caudal sections at the level of the midbrain, these radiated toward the retrosplenial cortex (RSPv Figures 11A,E,G) and additionally terminated in the subiculum (SUB, Figure 9G). More anteriorly, termination zones of cingulum bundle axons could be found in the anterior cingulate cortex (AC) (Figure 11F). These results were unchanged following spinal hemisection (data not shown), and agree with projections of the AV and AM nuclei demonstrated in the Allen Mouse Brain Connectivity Atlas.^1^

A consistent finding in Hoxb8-Cre^tdt^ mice was the presence of reporter-positive astrocytes (verified with Sox9 immunohistochemistry) (Sun et al., 2017) forming a distinct border between ventral midbrain and thalamus at the cephalic flexure (*CF*) (Figures 11A,H–J).

**Figure 12:**
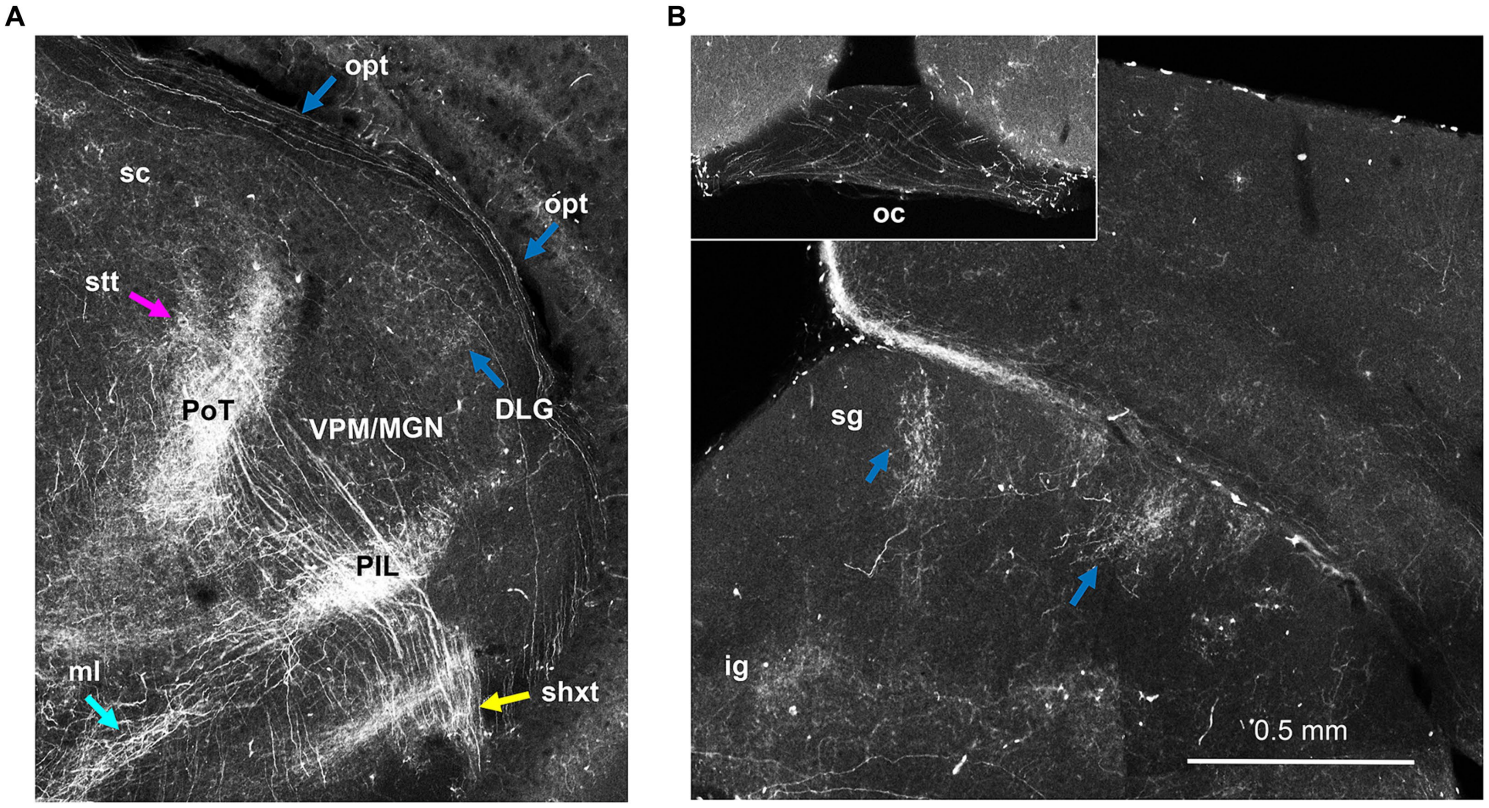
Sparse labeling of retinal ganglion cells in Hoxb8-Cre^tdt^ mice. **(A)** Thalamic section demonstrating axons in the optic tract (opt) and terminations in the dorsal lateral geniculate nucleus (DLG), just lateral to the rostro-caudal interface between the medial geniculate nucleus (MGN) and ventral posterior medial nucleus (VPN). **(B)** Sparsely-labeled axons decussate in the optic chiasm (inset). Opt axons also terminate as tufts in the superficial grey layer (sg) of the superior colliculus. ig, intermediate grey layer of the superior colliculus; medial lemniscus; PIL, posterior intralaminar nucleus; PoT, posterior triangular complex of the thalamus; shxt, spinofugal decussating tract; stt, spinothalamic tract; ZI, zona incerta.

As was the case for other scattered cells, a few recombined retinal ganglion cell axons were apparent in the optic tract (Figure 12A), and optic chiasm (Figure 12B, Inset). These followed the optic tract dorsally and terminated in the dorsal lateral geniculate nucleus of the thalamus, as well as in the superficial grey of the superior colliculus in discrete tufts (Figure 12B) – these were also unaffected by C3 hemiseciton (data not shown).

**Figure 13:**
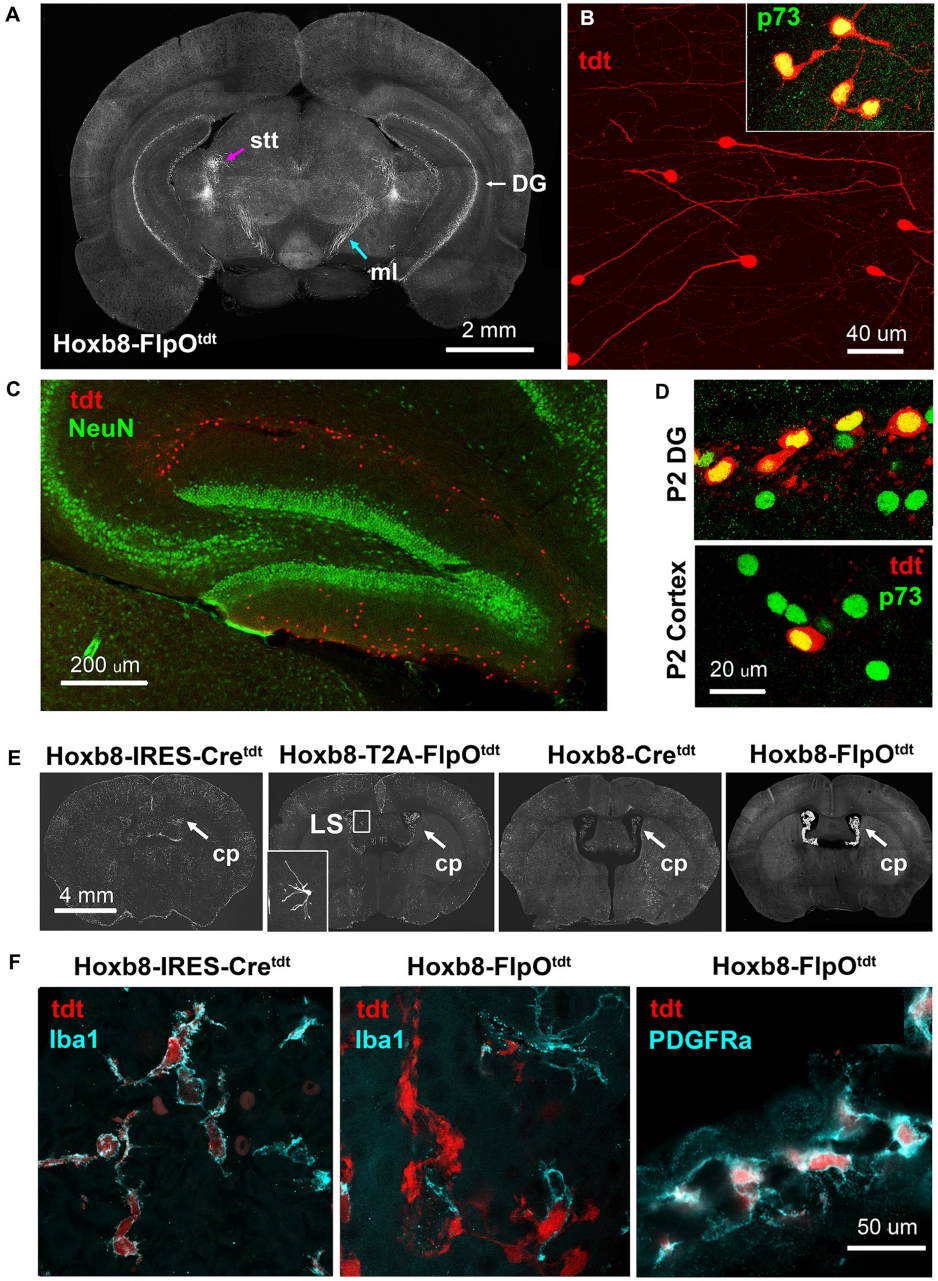
Ectopic Hoxb8-driven reporter expression in Hoxb8-FlpO^tdt^ mice. **(A)** Coronal section at the level of the caudal thalamus indicating the spinothalamic tract (stt) and medial lemniscus (ml), and the dentate gyrus. **(B)** High-power images of reporter-expressing dentate gyrus cells, and their expression of Cajal–Retzius-specific p73. **(C)** Cajal–Retzius cells are NeuN-negative. **(D)** In the P2 dentate gyrus, but not the P2 cortex, about 50% of Cajal–Retzius cells are also reporter-positive – reporter-positive cortical Cajal–Retzius cells are rare at this age. **(E)** Heavy reporter expression in the choroid plexus (cp) of Hoxb8-FlpO^tdt^ mice. Occasional neurons in the lateral septum of Hoxb8-T2A-FlpO^tdt^ mice had undergone recombination (inset). **(F)** In Hoxb8-IRES-Cre^tdt^ (and other) mice, reporter-expressing cells of the choroid plexus are Iba1-positive macrophages, whereas in in Hoxb8-FlpO^tdt^ mice, they are Iba1-negative, PDGRFa-positive mesenchymal fibroblasts.

In Hoxb8-FlpO^tdt^ mice, we identified a population of cells on the outer molecular layer of the dentate gyrus as late-surviving Cajal Retzius cells based on tadpole-like morphology and their expression of p73 (Figures 13A–D) (Anstötz and Maccaferri, 2020).

The choroid plexus of Hoxb8-FlpOtdt mice contained a dense population of tdtomato-positive PDGFRa-positive mesenchymal cells (fibroblasts) (Figures 13E,F). This contrasted with the other mouse lines, in which reporter-positive choroid plexus cells were of the monocytic lineage, expressing Iba1 (Figure 13F) and f4/80 (data not shown).

### 3.5 Microglia

Previous studies using Hoxb8 reporter mouse lines have highlighted the presence of Hoxb8-lineage microglia, thought to comprise one-third of all adult microglia in the mouse brain (Chen et al., 2010; Szabo et al., 2015; Bohic et al., 2023). Microglia were present in all Hoxb8 reporters assessed here, but differed markedly in abundance. Hoxb8-lineage microglia were most numerous in the cortex of Hoxb8-IRES-Cre^tdt^ mice followed by Hoxb8-Cre^tdt^ mice (Most readily apparent if Figures 8D, 10B,C). Interestingly, even fewer microglia were present in the other knock-in model (Hoxb8-T2A-FlpO^tdt^) (Bohic et al., 2023). There were almost no Hoxb8-lineage microglia in Hoxb8-FlpO^tdt^ mice.

## 4 Discussion

### 4.1 Towards a spinofugal projection atlas

The overarching aim of this study was to determine the feasibility of using Hoxb8 reporter mice to study the distal anatomy of spinofugal projections, with a longer-term goal of understanding their plasticity in the wake of neurotrauma such as spinal cord injury.

We have used the YSK Unified Anatomical Atlas^2^ (Chon et al., 2019) to map axons of spinal origin onto specific regions throughout the brain. This atlas was made in an effort to resolve discrepancies between the Allen Common Cordinate Framework (CCF) (Wang et al., 2020) and the Franklin-Paxinos mouse brain atlas (Paxinos and Franklin, 2019). Another issue is the relationship between axonal projections and cytoarchitectonically-defined nuclei: spinofugal projections are not necessarily overlaid onto the entirety of particular nuclei. The cerebellm is a case in point: subdivisions of spinofugal input are not apparent from gross granule cell cytoarchitecture, and therefore not included in standard atlases (Ozol and Hawkes, 1997; Gebre et al., 2012). Additionally, in more laminated structures such as the superior colliculus, dendrites (whiech receive the bulk of synaptic input) extend into distant layers. These findings beg the development of a spinofugal projection atlas; this work represents an important step in that direction.

The genetic labeling technique used here reveals the presence of a previously undescribed tract of axons arising from the spinal cord, decussating in the ventral hypothalamus, with the zona incerta as a likely zone of termination. Bulk lineage tracing also emphasizes less compact spinofugal tracts running through neuropil and not normally apparent using general myelin stains. This feature sheds light on a textbook “black box” that exists between axonal tracts in the spinal cord and their terminal fields in supraspinal structures. Figure 3 illustrates a bottleneck of ascending ventral and lateral tracts, anterior to which multiple ascending tracts diverge from the middle cerebellar peduncle (Figure 7). This point of defasciculation and/or branching occurs over just a few hundred microns in the rostro-caudal direction, and gives rise to at least five branches: a compact tract running caudally toward the cerebellar decussation (the inferior cerebellar peduncle proper), a diffuse dorsal tract of thick axons coursing rostrally toward the thalamus (the spinothalamic tract), a second diffuse spinotectal tract running dorsolaterally and dorsomedially with a prominent decussation in the superior colliculus, and two more diffuse tract projecting to the PAG (medially) and ECIC (laterally) in the inferior colliculus. The border between midbrain and hindbrain is established at this location early in development (the midbrain-hindbrain boundary, MHB, aka isthmus) and where an important fibroblast growth factor (FGF) and Wnt-secreting signaling centre emerges which patterns the surrounding tectum, pons, and cerebellum. These morphogens, in addition to dictating the fate of responsive cells, can act on growth cones to shape axonal trajectories (Charron and Tessier-Lavigne, 2005; Zou and Lyuksyutova, 2007). Hoxb8 reporter mice should serve as useful tools to track growth of spinofugal axon tracts during embryogenesis and their responses to manipulations perturbing isthmic signaling.

The presence of labeled axons in the medial lemniscus (ml), particularly in the non-targeted Hoxb8 reporters (Figures 5–7) was somewhat surprising. The ml is generally thought to consist only of post-synaptic projections from the dorsal column nuclei, which relay low threshold cutaneous and joint positional information to the thalamus. The absence of recombination above C4 in Hoxb8-Cre^tdt^ and Hoxb8-FlpO^tdt^ mice (and their degeneration following C3 hemisection in the Hoxb8-Cre^tdt^ mice) (Figures 3, 9) means that many of these axons arise from more caudal spinal neurons. This may represent either a population of post-synaptic dorsal column axons traversing the dorsal column nuclei (but remaining ipsilateral given their degeneration following hemisection, Figure 7) or, more likely, a divergence of ascending axons rostral to the anterolateral bottleneck in the medulla – our observations indicate that this occurs somewhere between the brainstem-spinal cord border and the rostroventral pons (possibly also at the MHB). The larger number of reporter positive axons in the ml of knock-in strains may additionally (or alternatively) reflect the presence of Hoxb8-lineage neurons in the gracile nucleus (Figure 3).

### 4.2 Regulation of Hox gene expression and implications of its dysregulation in lineage tracing

Many of the differences between targeted and non-targeted reporter lines were expected based on the differing rostral Hoxb8 expression boundaries (C4 in both non-targeted strains). Obvious examples include the relatively lower density of labeled axons projecting to the thalamus via the medial lemniscus in Hoxb8-Cre^tdt^ and Hoxb8-FlpO^tdt^ mice, and the absence of a projection from the nucleus of the solitary tract to the bed nucleus of the stria terminalis. These, and more striking occurrences of ectopic recombination in Hoxb8-Cre^tdt^ mice (anterior thalamic nuclei, astrocytes beneath the cephalic flexure, retinal ganglion and vomeronasal axons) and Hoxb8-FlpO^tdt^ mice (Cajal–Retzius cells in the dentate gyrus, choroid plexus fibroblasts) can most easily be explained by the absence of important negative regulatory elements in the transgene constructs and resulting sensitivity to nearby sources of diffusible signaling molecules during development (more on this below). Other differences, particularly between mice in which recombinase was targeted to the native Hoxb8 locus, are more difficult to reconcile. For example, there was an obvious difference in reporter-positive microglial density between Hoxb8-IRES-Cre^tdt^ and Hoxb8-T2A-FlpO^tdt^ mice. The presence of tdtomato-positive axons in the core of the facial motor nucleus in Hoxb8-IRES-Cre^tdt^ mice only, may also speak to artefactually elevated regulation of Hoxb8 protein expression. As such, some discussion of Hox gene regulation under normal circumstances is warranted.

Let us first consider the circumstances in non-targeted strains, in which the extent of reporter expression is simultaneously absent from tissues normally expressing Hoxb8 (e.g., the upper cervical cord), and expressed ectopically in more rostral structures. The utility of random transgene insertions to report transcriptional activity of a given gene is affected (either positively or negatively, depending on the question being asked) by the absence of regulatory elements controlling endogenous expression. In the case of the construct used to generate Hoxb8-Cre and Hoxb8-FlpO mice, the transgenes are no longer in the vicinity of an important retinoic acid response element (RARE) called the distal element (DE), positioned nearby between the Hoxb4 and Hoxb5 genes (Oosterveen et al., 2003; Valarché et al., 2003; Afzal et al., 2023), which is responsible for extension Hoxb8 expression from C4 to the obex.

Ectopic recombination, on the other hand, could be the result of the absence of suppressive regulatory sequences, insertion of the transgene in the vicinity of non-canonical promoters or enhancers, or temporally-altered chromatin accessibility. Whichever mechanism (or combination of mechanisms) is responsible, it still requires a stimulus for the activation of Hoxb8 expression. The dependence of Hoxb8 transcription on retinoic acid, elegantly dissected by the Deschamps and Krumlauf groups (Charité et al., 1995; Bel-Vialar et al., 2002; Forlani et al., 2003; Afzal et al., 2023), makes developmental exposure to this diffusible signaling molecule a likely cause. Indeed, ectopic recombination occurred close to tissues expressing retinoic synthesizing enzymes (RALDH1 and RALDH2) (olfactory epithelium, retina, craniofacial mesenchyme below the cephalic flexure), choroid plexus [Ruberte et al., 1993; Yamamoto et al., 1998; Luo et al., 2004 and atlas.brain-map.org], and/or in brain regions rendered sensitive to endogenous retinoic acid using a RARE-driven LacZ reporter (anterior thalamic nuclei, dentate gyrus) (Luo et al., 2004). If RA is the responsible driver of ectopic recombination, then timing of transgene RARE accessibility is most likely to underpin differences between Hoxb8-Cre and Hoxb8-FlpO expression.

Retinoic acid-dependent Hox gene regulation has many layers, and relief from Hoxb8 suppression must contribute to ectopic expression. Recently, Afzal et al. (2023), used CRISPR/Cas9 to generate mutations in the DE and two other RAREs (B4U and ENE) flanking the Hoxb4 coding sequence to determine their role in Hoxb regulation. With DE and ENE mutations, there was increased transcription of both Hoxb9 and Hoxb1, with a greater increase in the ENE mutant embryos (Afzal et al., 2023). The B4U mutation did not result in changes from wild-type mice with respect to Hoxb9 or Hoxb1 transcription, but Hoxb4 transcription was increased. This suggests that the DE and ENE are important in the regulation of all Hoxb genes, while B4U acts on more closely adjacent sequences in the Hoxb cluster (Afzal et al., 2023). Given that loss of control by ENE led to an increase in Hoxb9 transcription, its absence may similarly affect the Hoxb8 transgene leading to its ectopic expression.

Non-targeted reporter mice also lack a miRNA target sequence (specific for miR-196a) in the 3’ UTR of the transgene, required for restricting anterior Hoxb8 expression (Yekta et al., 2004; Hornstein et al., 2005). Without it, and under the influence of appropriate Hoxb8-inducing signals, one might expect transgene expression rostral to the brainstem.

Neither of the targeted recombinase models is expected to interfere with Hoxb8 function, and while Cre and FlpO have equivalent excision efficiencies (Birling et al., 2012), subtle differences in transcriptional or translational control might be anticipated: whereas the IRES system results in less efficient transcription of the second gene in the sequence [20–50% of the first, (Mizuguchi et al., 2000)], the near 100% efficiency of T2A self-cleavage should result in a 1:1 ratio of Hoxb8 and FlpO recombinase protein. The IRES has been reported to protect flanking sequences from miRNA-mediated inhibition of translation (Humphreys et al., 2005; Mathonnet et al., 2007; Thermann and Hentze, 2007). If this is the case for Hoxb8-IRES-Cre mice, then it may be considered a Hoxb8 over-expressor, potentially explaining the abundance of Hoxb8-lineage microglia and the unique presence of spinofugal projections to the facial nucleus’ core (Figure 3).

The differences between the four reporter strains described here raise questions about the fidelity of permanent reporter expression to endogenous Hoxb8 expression. The two targeted transgenic (knock-in) models are a case in point: there was an obvious difference in the number of reporter-positive microglia (Hoxb8-IRES-Cre^tdt^ > > Hoxb8-T2A-FlpO^tdt^), and lateral septal neurons were only apparent in Hoxb8T2AFlpO mice (Bohic et al., 2023). One hypothesis is that this is a result of the relationship between differing lengths of the knocked-in constructs and the timing of Hox gene expression (i.e., temporal collinearity or the “Hox clock”). Increased genomic distance between the Hoxb8 sequence and the mir-196a target in the 3’UTR may result in a prolonged expression of Hoxb8 and evasion of microRNA-mediated mRNA degradation.

An approach to determining the true distribution of Hoxb8 expression might be to identify a sequence specifically transcribed in response to Hoxb8, and construct a reporter driven by its promoter. A first step towards this goal is identification of genes upregulated by Hoxb8 overexpression, and has recently been accomplished in the chick (Wilmerding et al., 2021).

Hoxb8-driven reporter expression is a viable approach to map axonal projections from the spinal cord to the brain. It offers superb visualization of trajectories otherwise inferred (spinothalamic and spinotectal tracts) or previously unknown (spinofugal hypothalamic decussating tract). Differences in transgenic constructs also provide some information on somatotopy (i.e., projections from the whole cord or from that up to C4 only). Models of choice for defining ascending pathways are the FlpO lines, given the relative paucity of Hoxb8-lineage microglia in both strains, and spatial separation of ascending pathways from the dentate gyrus in the Hoxb8-FlpO line.

## Data availability statement

The original contributions presented in the study are included in the article/Supplementary material, further inquiries can be directed to the corresponding author.

## Ethics statement

The animal study was approved by the University of British Columbia Animal Care Committee. The study was conducted in accordance with the local legislation and institutional requirements.

## Author contributions

BB: Data curation, Investigation, Methodology, Visualization, Writing – original draft, Writing – review & editing. WS: Formal analysis, Investigation, Visualization, Writing – review & editing. MB: Resources, Writing – review & editing. AU: Resources, Writing – review & editing. VA: Funding acquisition, Resources, Writing – review & editing. MR: Conceptualization, Funding acquisition, Investigation, Project administration, Supervision, Visualization, Writing – original draft, Writing – review & editing.

## Footnotes

^1^https://connectivity.brain-map.org/

^2^https://kimlab.io/brain-map/atlas/

## Supplementary material

Supplementary Video S1 Lightsheet imaging of the superior colliculus and rostral inferior colliculus in a Hoxb8-Cre^tdt^ mouse. The sequence of images begins dorsally. Note the meshwork of projections in the intermediate grey of the superior colliculus, and deeper axons decussating near the superior/inferior collicular border. Best seen at 2x playback speed.

Supplementary Video S2 Light sheet imaging of the thalamus at the level of the VPL nucleus of the thalamus (lateral meshworks), and the anterior ventral and anterior medial nuclei of the thalamus (medial dense clusters) four weeks following lateral hemisection (right side in the video) in a Hoxb8-Cre^tdt^ mouse.
